# Effects of Touchscreen Media Use on Toddlers’ Sleep: Insights from Longtime ECG Monitoring

**DOI:** 10.3390/s21227515

**Published:** 2021-11-12

**Authors:** Sigrid Hackl-Wimmer, Marina Tanja Waltraud Eglmaier, Lars Eichen, Karoline Rettenbacher, Daniel Macher, Catherine Walter-Laager, Helmut Karl Lackner, Ilona Papousek, Manuela Paechter

**Affiliations:** 1 Educational Psychology Unit, Department of Psychology, University of Graz, 8010 Graz, Austria; marina.eglmaier@uni-graz.at (M.T.W.E.); daniel.macher@uni-graz.at (D.M.); manuela.paechter@uni-graz.at (M.P.); 2 Department of Early Childhood Education, University of Graz, 8010 Graz, Austria; lars.eichen@uni-graz.at (L.E.); karoline.rettenbacher@uni-graz.at (K.R.); catherine.walter-laager@uni-graz.at (C.W.-L.); 3 Otto Loewi Research Center, Division of Physiology, Medical University of Graz, 8010 Graz, Austria; 4 Institute of Medical Engineering, Graz University of Technology, 8010 Graz, Austria; 5 Biological Psychology Unit, Department of Psychology, University of Graz, 8010 Graz, Austria; ilona.papousek@uni-graz.at

**Keywords:** wearable biomedical sensing, heart rate, heart rate variability, ECG derived respiration, sleep latency, restless sleep, 3D acceleration sensor

## Abstract

Wearable biomedical sensor technology enables reliable monitoring of physiological data, even in very young children. The purpose of the present study was to develop algorithms for gaining valid physiological indicators of sleep quality in toddlers, using data from an undisturbing and easy-to-use wearable device. The study further reports the application of this technique to the investigation of potential impacts of early touchscreen media use. Toddlers’ touchscreen media use is of strong interest for parents, educators, and researchers. Mostly, negative effects of media use are assumed, among them, disturbances of sleep and impairments of learning and development. In 55 toddlers (32 girls, 23 boys; 27.4 ± 4.9 months; range: 16–37 months), ECG monitoring was conducted for a period of 30 (±3) h. Parents were asked about their children’s touchscreen media use and they rated their children’s sleep quality. The use of touchscreen media predicted the physiologically determined quality of sleep but not parent-reported sleep quality (such as sleep onset latency). Greater heart rate differences between restless sleep phases and restful sleep indicated poorer nighttime recovery in children with more frequent use of touchscreen media. The study demonstrates that the expert analysis of the ECG during sleep is a potent tool for the estimation of sleep quality in toddlers.

## 1. Introduction

Advances in sensing technology have produced very small and light devices, while upholding measurement accuracy. These portable sensor devices—or wearables—have been advancing health-related data collections in clinical trials and research tremendously over the past years [1]. They simplify implementation, enable continuous monitoring in everyday-life situations, and might (to some extent) even increase compliance of participants, as they are barely perceptible and collect the data passively.

These achievements also open up new opportunities for research on the development of very young children. Monitoring infants and toddlers can support research in areas in which effects of certain conditions on development are still unknown. Such an area is the use of digital media by young children and the effects of media use on development. Portable sensor devices can be used during sleep, in which processes of learning and memory consolidation [2,3], as well as neurodevelopment, take place [4,5]. Furthermore, they may help one to understand children’s perceptions of digital media use, and might, in the long run, explain its effects on developmental processes. The present cross-disciplinary study is aimed at developing suitable algorithms for the analysis of data gained by wearable biomedical sensing technology in children, and to report the application of this technique—in investigating the impact of touchscreen media use on the quality of sleep of toddlers.

Since technical equipment in industrialized households has increased [6], various digital media devices or new media have become accessible to even very young children [7,8]. Especially touchscreen devices like tablets and smartphones attract much attention. They enable interactive and versatile use, are portable (thus convenient and readily available), and their sensory stimuli arouse interest [9,10]. Risks and benefits of the use of touchscreen media by young children are being debated. Although extended (touch)screen use may have detrimental effects on the well-being of children of all ages (for an overview see [11]), media use does not necessarily have unfavorable effects. Moderators include age-appropriate use and content [12,13], adult accompaniment [14], and parental mediation [15]. Nevertheless, there is an increasing number of studies reporting on the adverse effects of digital media use in young children’s sleep e.g., [16,17,18,19]. Since, to date, these studies have relied only on parental reports, it seems inevitable to introduce objective measures on children’s sleep into this field of research.

Young children’s sleep may be affected by various factors, such as inefficient bedtime routines [20], family background [18], health status [21], and perhaps digital media use [16,17,18,19]. Sleep is crucial for infants and toddlers, as it is vital for many developmental processes (for an overview see [22]). These comprise brain- and neurodevelopment [4,5,23], cognitive development [2,3,23], as well as physical growth [24,25]. Throughout infancy, children spend approximately half of the day sleeping. According to the national sleep foundation, the recommended hours of sleep (per 24 h) are: for infants (1–11 months) 12 to 15 h, for toddlers (1–2 years) 11 to 14 h, and for preschoolers (3–5 years) 10 to 13 h [26]. Understandably, too little or poor sleep is associated with detrimental effects on children’s health and well-being [20]. 

Considering 24 h in a toddler’s life, they are largely filled with sleep and naptimes, several mealtimes, time for personal hygiene, and in some cases, time spent in a daycare. Hence, only a few hours remain for playing, jaunting, or doing other things together with parents. The extra time spent on media use may, to some extent, come at the expense of playing [6] or the duration of sleep at night (*sleep time* or *nighttime sleep*) [16,17,19,27], and may increase the time it takes to fall asleep (*sleep onset latency*), which, in turn, is assumed to reduce the sleep time [17,18,28]. The overall duration of sleep at night or the total sleep duration (usually the sum of day and night sleep) provides information on (in)appropriate time for regeneration. Whereas in adults, sufficient sleep benefits cognitive performance and recovery [29], it is assumed to stimulate cognitive development in children [22]. As there is not one fixed definition for the quality of sleep, several variables—such as *sleep time*, *nighttime sleep*, and *sleep onset latency*—can be used to estimate sleep quality [30].

Subjective data, such as questionnaires or sleep-wake-diaries, do not describe sleep quality satisfactorily, and should be complemented by objective data derived from actigraphy [31], polysomnography (PSG) [32], or electrocardiography (ECG), and respiration [33,34]. Whereas PSG is usually regarded as the “gold standard assessment of sleep behaviors” [27] (p. 15), it may not be suitable for every purpose [34,35]. The technically sophisticated and elaborate method seems more appropriate for clinical surveillance or diagnostics of sleep disorders than for the estimation of a person’s regular sleep quality, as the technical equipment and unfamiliar environment may disturb the sleep considerably. This is, of course, of even more importance in children. Moreover, recent studies question if PSG may detect the full scope of sleep patterns for children [36], and point to problems of feasibility of laboratory studies in young children [27]. By use of portable ECG devices, undesired interfering effects of sleeping with many uncomfortable electrodes, and in an unfamiliar setting, can be avoided. Moreover, new generations of Holter devices, such as little obtrusive wearable sensors or adhesive patch monitors, provide high precision in data recording, despite using only one single lead [37]. Therefore, less obstructive monitoring does not need to be exchanged for loss in data quality. High-quality non-invasive technology that requires the application of one or two skin patches only is an attractive option, particularly for the examination of young children. Data are collected passively and efficiently [1]. At the same time, the little burdensome attachment of the equipment and unrestricted mobility while wearing it, increases participant outreach. ECG data, while not the most common physiological signals used in the analysis of sleep behaviors, provide information about the interaction between sympathetic and parasympathetic activities in the autonomic nervous system that reveal important information about a range of physiological states [38], and, as such, can deliver additional relevant information that may not be available from central nervous system variables. Finally, wearable sensing technology keeps costs and efforts manageable.

To our knowledge, few studies have used physiological methods, such as actigraphy, to investigate young children’s sleep; however, these studies focused on overall sleep composites [31] or sleep problems [39]. In the present study, we use data from portable ECG devices to analyze toddlers’ sleep and study the relationship between sleep quality and toddlers’ use of digital media, whereby we focus on touchscreen media only (smartphone and tablet). To consider the regenerative aspect of sleep, recordings were divided into restful and restless phases (for details see the Materials and Methods section). Whereas restful sleep corresponds to untroubled sleep, restless sleep has little restorative potential, and is often related to sleep disorders [21]. Thus, relative differences between phases of restful and restless sleep may provide relevant information in the context of this study. 

Taken together, the aims of this study are:

- To develop suitable methods to gain physiological indicators of sleep quality in young children from ECG data, which were recorded with wearable, undisturbing devices.
- These novel methods are applied in a highly topical context, that is, to study the impact of touchscreen media use on the quality of sleep in toddlers; thus, obtaining indication of their practical relevance.

## 2. Materials and Methods

### 2.1. Participants

Parents and their children were recruited at daycare centers. First, local carrier associations were informed about the study, which was announced as a study on the effects of toddlers’ use of digital media, including long-term ECG monitoring. After positive feedback, daycare centers were contacted, which were equally located in urban and rural areas. In the selected daycare centers, the aims and procedure of the study were presented to the early childhood educators and parents. Parents agreed to participate in the study on a voluntary basis. Inclusion criteria were regular attendance of institutional daycare and appropriate age (approximately 1–4 yrs.). Exclusion criteria were reports of cardiovascular or neurological disease and premature birth (i.e., less than 37 weeks of gestation).

A total of 71 parents and their young children participated in the study. Of these, two parents refused the recording of the ECG in their children and handed in the questionnaires only. Of the remaining 69 participants, a further 14 children were excluded due to low quality or insufficient recording time of the ECG (two devices were switched off too early, six recordings were stopped by the parents before bedtime or did not comprise the full night, and data of six children included too many artefacts for further analyses). Thus, the final analyses referred to 55 toddlers (32 girls, 23 boys; 27.4 ± 4.9 months; range: 16–37 months).

The ECG recordings took place from Monday to Thursday between 07:30 and 09:00 a.m., and continued into the afternoon of the following day (i.e., between 01:30 and 04:00 p.m. the next day). Therefore, the monitoring resulted in 30 to 33 h of ECG data for each toddler, including an entire night.

Each parent gave written informed consent to participate in the study and to provide data about themselves and child. The study was performed in accordance with the 1964 Declaration of Helsinki and was approved by the authorized local ethics committee (39/52/63 ex 2017/18; 28 May 2018).

### 2.2. Acquisition of ParentReported Data

The questionnaire for parents was completed by the toddlers’ biological parents (50 mothers, 4 fathers, 1 missing). Items included information about their age (35.01 ± 5.9 years; *n* = 54), media equipment at home, and net household income per month (answers per category: 3 answers for “up to EUR 2000“; 11 for “EUR 2000 to EUR 3000“; 20 for “EUR 3000 to EUR 4000“; 7 for “EUR 4000 to EUR 5000“; 4 for “over EUR 5000“; 9 for “I do not want to provide that information”; 1 missing). Furthermore, they were asked about their child’s gender and date of birth (to calculate the exact age at the day of the ECG recording), and were asked to report about their child’s media use and sleep habits.

#### 2.2.1. Sleep Habits and Quality of Sleep

To gather parentreported information on the toddlers’ quality of sleep, two variables were collected. The corresponding questions are presented in parentheses. Means and standard deviations are presented in Table 1:

- *Nighttime sleep* (how many hours did your child usually sleep at night within the last 4 weeks?)
- *Sleep onset latency* (how long did it usually take for your child to fall asleep, in the evenings, on average, within the last 4 weeks?)

In addition, parents and early childhood educators were asked to continuously record each participating child’s activities (especially sleep- and naptimes, mealtimes, and activities, such as playing) in an activity log, including the day, the corresponding time, duration of the activity (i.e., the start and end time of an activity), and the physical position (sitting, lying, or upright position). The adult reports in this activity log yielded the variable *sleep time*, which represented a toddler’s hours of bedtime during the very night of the ECG recording (see also Table 1).

**Figure 1:**
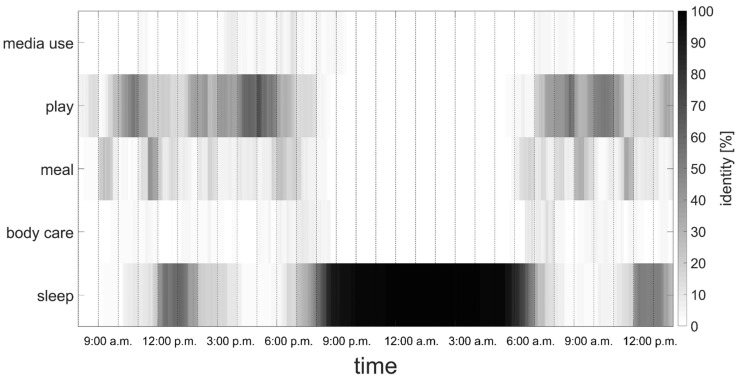
Representation of a typical day of the toddlers, all attending a daycare. The color code shows the nature of the activities of all toddlers; that is, the color black indicates that all toddlers “did” the same, at the specific time of day. The figure demonstrates that a toddler’s everyday life is well structured, especially in terms of sleeping times, but there is no “specific hour for media use”.

Furthermore, the recorded activities during the ECG monitoring were coded hierarchically; that is, e.g., at the lowest level, *brush teeth*, *bath*, *put clothes on*, and in a broader category *body care*. As a second example, *breakfast*, *lunch,* and *dinner* are different categories at the lowest levels, but all belong to the main category *meal* (see Figure 1). In addition, all subcategories were separated into active (e.g., walking) and passive activities (e.g., car ride). On average, 85.2 ± 8.6% of the ECG monitoring times had records of activity in the activity log. The detailed information was used to evaluate the sleep quality algorithm (see Section 2.3.1).

#### 2.2.2. Toddlers’ Media Use

According to the parental reports, the households were well equipped with digital media (e.g., internet: 51 yes, 3 no, 1 missing; smartphones: 54 yes, 1 missing; tablet: 42 yes, 12 no, 1 missing; PC/computer/laptop and TV each: 52 yes, 2 no, 1 missing), similar to reports in previous studies [6,16]. Furthermore, parents were asked about how long their children use various media on weekdays as well as on weekends (ranging from “0 = never”, “1 = less than one hour per day”, “2 = more than one hour per day”, “3 = more than two hours per day” to “4 = more than three hours per day”). The questionnaire comprised several types of media, e.g., smartphone, tablet, television, audio media, computer/PC/laptop. Media use in our sample was lower than in other studies, e.g., [17], especially because some children did not use any digital media at all. In detail, we found that 29 of the toddlers ever use a smartphone and that only 16 out of 55 children have regular access to a tablet. Table 2 displays the overall frequencies of toddlers’ media use.

In the present paper, we focus on touchscreen media only, i.e., smartphones and tablets. In accordance with other studies on the effects of screen time, e.g., [19,40], an average score of weekly media use was calculated; that is, the weighted mean of daily screen times for a smartphone and tablet: ((weekdaysmartphone × 5) + (weekendsmartphone × 2) + (weekdaytablet × 5) + (weekendtablet × 2))/7.

### 2.3. Acquisition and Processing of ECG Data

The ECG was recorded continuously using a portable sensing device (eMotion Faros 180°, Bittium Biosignals Ltd., Kuopio, Finland; single-channel ECG, using Einthoven Lead II set-up, with a sampling rate of 1 kHz; weight: 13 g; size: 48 × 29 × 12 mm). The devices also featured a 3D acceleration sensor that provided continuous information on the activity level. For the analysis, all raw data were converted from EDF to a MATLAB^®^ data format. In brief, to check the quality of the ECG data and to calculate the interbeat interval time series, we used a semi-automatic artifact handling protocol developed and used by our research group for several years [38,41]. The respiratory rate was determined offline using ECG derived respiration [42]. Details of our data preprocessing routine are reported in Lackner et al. [38].

#### 2.3.1. Quantification of Sleep Quality

ECG data, the data from the 3D acceleration sensor as well as the activity log, were used to determine an index of being in a resting state to assess the sleep quality. Variables were calculated for segments of two minutes with a step size of 30 s, i.e., for a recording period of 24 h this results in a total of 2880 data points for each variable. The determination of indicators for sleep quality requires several steps of preprocessing.

Physiological variables cannot only provide information on sleep stages, such as REM-sleep or non-REM-sleep [43], but also display information on the regenerative potential of the sleep. Whereas restful sleep is indicated by a long sleep duration [29], several deep sleep phases, and only few awakenings, restless sleep shows few deep sleep phases and a high frequency of night awakenings in relation to the overall sleep duration and, is, therefore, at the expense of regeneration and recovery [44]. For each toddler, sleep was divided into restful and restless phases.

The algorithm for determining the phases for “restless” and “restful” sleep is based on the following criteria:

- Duration of sleep according to the activity log: plausibility of this information was checked based on the activity data from the 3D acceleration sensor (position detection and variance of the signal) as well as the corresponding heart rate change.
- Validity and variance of the data from the 3D acceleration sensor: a 2-min segment is marked as representative of a “resting period” when the variances of the acceleration signals fall below an empirically determined threshold. Individual limits were determined based on the variance distributions of the sensors during the 32 h period of data collection, and evaluation of their sensitivity and specificity by using, e.g., entries in the activity log, such as “nap” or “afternoon nap”).
- Validity of ECG and EDR (ECG-derived respiration): a 2-min segment is marked as representative of a “resting period” when a valid determination of the respiration pattern in the ECG signal is possible for an empirically determined proportion of data (due to the given population of toddlers this threshold was set to two thirds).
- Validity of ECG and the quotient of heart rate variability (HRV; using the time domain parameters SDNN and RMSSD): the interaction of the two branches (sympathetic and parasympathetic) of the autonomic nervous system was used as an indicator for activity or rest. Individual limits were determined based on the variance distributions of the variable during the 32 h period of data collection, and evaluation of their sensitivity and specificity by using, e.g., entries in the activity log, such as “nap” or “afternoon nap”).
- These criteria for the determination of the 2-min “resting periods” must be fulfilled for at least 10 consecutive minutes, to fully meet the “rest” criterion.

**Figure A1:**
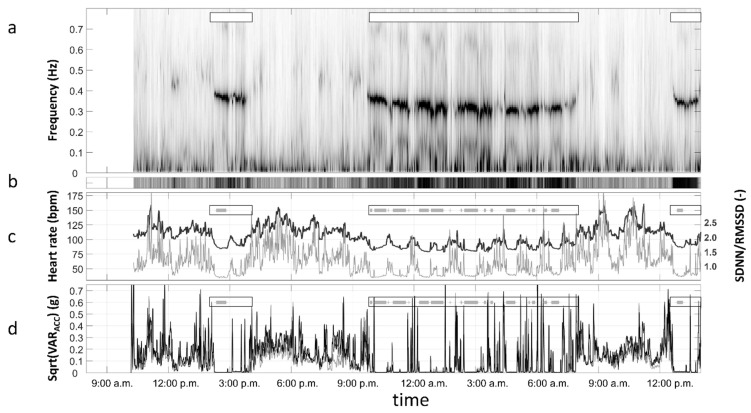
Graphical representation of the data analysis of one exemplary participant. (**a**) Spectrogram of the R–R interval: the power spectral density for segments of two minutes with a step size of 30 s; the color black indicates maximal power. The white colored bars on the upper part show the “afternoon nap” from 2:00–4:00 p.m., the night sleep period from 9:45 p.m.–8:00 a.m., and the “nap” from 12:15–1:45 p.m., at the second day of recording). (**b**) Percentage of valid determination of the respiration pattern in the ECG signal; the color black indicates 100% (white: 0%). (**c**) Heart rate (black) and the ratio SDNN/RMSSD (gray) over time. (**d**) Square root of the variance of the 3D-ACC over time. The gray bars in (**c**,**d**) within the white colored bars show the detected phases of restful sleep. It can be seen that restful sleep phases can be detected not only in the night, but during the “nap” and “afternoon nap” as well, but due to the strict selection criteria not during the rest of the day.

To evaluate the algorithm in terms of *false positive* “restful” sleep phases, the whole 30 to 33 h of ECG data were analyzed. On average, 79.73 ± 13.46% of “restful” sleep phases were detected in sleep, 18.39 ± 12.86% in sleep periods during the day. The detection showed 0.76 ± 2.24% *false positive* “restful” sleep phases during other activities (such as “car ride on the way home from the daycare center”) and 1.11 ± 2.12% in undocumented time periods during the day. That is, on average less than 2% were detected as *false positive*. For further technical details, see Figure A1 and the description in Appendix A.

The following variables for the quality of sleep were calculated (Table 3):

- *dHR sleep*—average heart rate during restless sleep periods minus average heart rate during restful sleep periods (beats per minutes, bpm);
- *Sleep duration*—duration of sleep at night based on the ECG data (hours, see above, slightly corrected, if necessary, according to the activity log).

On average, the HR during restful sleep was 94 bpm (*M* = 93.54, *SD* = 10.46), during restless sleep 101 bpm (*M* = 101.02, *SD* = 11.12).

### 2.4. Study Design and Procedure

Data acquisition was implemented in a field study, to make it as convenient and simple for parents and their toddlers as possible. We started the ECG long-time recordings early in the morning at the daycares. As soon as the young participants arrived with their parents, they were equipped with the ECG recorder by the researcher in charge, and parents received the questionnaire and the activity log. All parents received instructions for the handling of the recorder (e.g., use of close-fitting clothing for the child to keep the electrodes attached to the body; the child could do everything, but it was advised that the recorder should not get in contact with water; parents were advised to only take the recorder off their child if absolutely necessary, and if possible, reinstall it again; the recording was non-invasive and harmless). Parents and educators were asked—at best—to not intervene or direct attention to the ECG recorder, to prevent disturbance of the passive data collection.

Educators and parents were instructed to take off the ECG recorder in the afternoon of the following day.

## 3. Results

### 3.1. Prediction of the Quality of Sleep

To analyze if the frequency of media use can predict toddlers’ quality of sleep, two standard multiple regression analyses were calculated. Regression analyses fit a model on the data to predict the manifestation of an outcome variable [45]. The regression analyses also included sociodemographic variables for control, in order to ensure that effects were independent from potential influences of age and gender (see also [17,19]). The outcome variables were either *sleep duration* or *dHR sleep* (objective physiological variable indicating quality of sleep). Additional variables were not used in the analyses.

The first regression analysis using *sleep duration* as the dependent variable did not yield a significant result (F(3,47) = 0.656, *p* = 0.583, *R*^2^ = 0.04). That is, taken together, the analysis did not show an influence of the independent variables (*touchscreen media use, age, gender*) on *sleep duration*. However, regressing the second outcome variable, *dHR sleep* on the variables *age*, *gender*, and *touchscreen media use* (F(3,47) = 4.936, *p* = 0.005, *R*^2^ = 0.24) revealed significant relationships between *touchscreen media use* and *dHR sleep* (*β* = 0.325, *p* = 0.014) and between *age* and *dHR sleep* (*β* = −0.378, *p* = 0.007). Statistical details are presented in Table 4. The regression coefficient *B* indicates the unique contribution of a specific predictor to the variance of the dependent variable, given in raw scores. For example, for each point, the degree of *touchscreen media use* becomes higher (e.g., from 1 = “less than one hour per day” to 2 = “more than one hour per day”, the difference between the heart rate during restless and restful sleep periods (*dHR*) becomes 1.286 beats per minutes higher. This increase in *dHR* is the estimated unique influence of *touchscreen media use*; that is, the variance of *touchscreen media use* that overlaps with other predictors (*age, gender*) is not included. The standardized regression coefficient *β* carries the same information as *B*, but uses the standard deviation as the (standardized) unit. It specifies how many standard deviations the dependent variable becomes higher (positive sign of *β*) or lower (negative sign), if the predictor increases by one standard deviation. For example, if the influences of all other predictors are held constant, for each standard deviation the degree of *touchscreen media use* becomes higher, the difference between the heart rate during restless and restful sleep periods (*dHR*) becomes 0.325 standard deviations higher (Table 4). The standard error (*SE*) is an estimator of the accuracy of prediction, and is given for more complete information, of more advanced users of statistics. The *p*-values indicate which of the predictors are considered to significantly (in the statistical sense) affect the dependent variable, independently of all other predictors (*p* < 0.05). Thus, the *p*-values indicate that, independent from age and gender, the degree of toddlers’ touchscreen media use impacts the restfulness of sleep *(dHR*; *p* = 0.014) but not sleep duration (*p* = 0.546).

To examine the effects on HR, we repeated these regression analyses, replacing *dHR sleep* with *HR restless sleep* (i.e., absolute HR during restless sleep) (F(3,47) = 3.954, *p* = 0.014, *R*^2^ = 0.20). This revealed the same relationship between *HR restless sleep* and *touchscreen media use* as above (β = 0.391, *p* = 0.004). When using the parent-reported variables of sleep quality, such as *sleep time* (F(3,47) = 1.003, *p* = 0.400, *R*^2^ = 0.06), *sleep onset latency* (F(3,47) = 2.156, *p* = 0.106, *R*^2^ = 0.12), or *nighttime sleep* (F(3,46) = 0.237, *p* = 0.870, *R*^2^ = 0.02) as dependent variables, no significant results were observed.

### 3.2. Relationships between Parent-Reported and Objective Data of Sleep Quality

Table 5 displays bivariate correlations between all study variables. A substantial correlation was observed between the physiologically-based variable *sleep duration* and the parent-reported variable *sleep time* (*r* = 0.867; *p* < 0.001). The correlation between *nighttime sleep* (parent-reported) and *sleep duration* (physiologically-based) was non-significant (*r* = 0.152; *p* = 0.278). The second physiologically-based variable, *dHR sleep,* did not correlate with any of the parent-reported variables of sleep quality, indicating that this variable pictures a different aspect of sleep quality than the mere duration of sleeping hours or hours spent in bed.

## 4. Discussion

The present study aimed to develop algorithms for the analysis of data gained by wearable biomedical sensing technology in children, with the purpose of gaining valid physiological indicators of sleep quality in an easy and undisturbing way, even in toddlers, which can then be applied in topical contexts, such as the impact of early touchscreen media use. In particular, the findings show high correspondence between the physiologically determined and parent-reported estimates of sleep duration, and highlight the validity of heart rate differences between restless and restful sleep phases as a presumed indicator of nighttime recovery. Nighttime recovery is of particular importance, so far, as it is not only a matter of well-being; the recovery process during restful sleep is assumed to stimulate cognitive development in children [22].

In the particular context of early touchscreen media use, the present findings demonstrated an advantage of the sleep quality variable that was based on physiological data compared to ratings provided by the toddlers’ parents. While touchscreen media use was not related to any of the parent-reported variables of sleep duration and quality, more extensive use of touchscreen media was associated with poorer nighttime recovery as indicated by greater heart rates during restless compared to restful sleep phases (*dHR*).

This finding corroborates previous indications of adverse effects of touchscreen media use on sleep quality [16,17,18,19]. The enhanced heart rate during phases of restless sleep in toddlers with more extensive media use may correspond to higher arousal ratings that so far were reported by subjective reports only [17,18]. Touchscreen media use did not predict sleep duration. This may be unexpected, because delayed sleep and reduced night time sleep are among the most frequently reported outcomes of extensive media use (for a review, see [46]). One possible explanation for this null result can be excluded: since, in the present study, touchscreen media use did not correlate with the parent-reported estimate of sleep duration either, the hypothesis that positive findings in the literature were due to some systematic bias in parental reportings can be rejected. A potential reason for the discrepant finding may be the narrow scope of the present study in terms of media use: while most other studies included various types of media, the present study focused on touchscreen media only. Related to that, there were only few reports of excessive media use in our sample (such as more than two hours of watching TV every day; see Table 2). On the other hand, toddlers in our sample had highly invariable and consistently age-appropriate sleep times. 

In addition, it is noteworthy that the physiologically estimated restfulness of sleep (*dHR*), but not any of the parent-reported variables, showed relationships to toddlers’ touchscreen media use. The most likely explanation for that is that none of the parent-reported variables captured a quality of sleep criterion similar to *dHR*, and the degree of touchscreen media use at the toddler’s age affects the restfulness of sleep in particular. This is plausible insofar, as, for instance, the time spent in bed is to a very high degree prescribed by the parents. Consequently, there is hardly any variance left in these variables that could be influenced by the toddler’s media use. Moreover, the null findings may underline the limits of subjective data when complex relationships are in the focus of interest. Sleep onset latency, in particular, which was reported to be affected by media use in previous studies [17,18], may be difficult for parents to rate. In the course of young children’s development, sleep patterns may vary across nights; thus, parental reports may not be representative. A recent study concluded that sleep onset latency does not represent common sleep dynamics in infants and, thus, even excludes this variable from the list of most representative variables of infants’ sleep [31].

### 4.1. The Use of Wearable Biomedical Sensing Technology in Very Young Children

Wearable sensing technology is a promising tool as small potent devices are now available at a large scale. They do not only allow gaining objective data; continuous passive recording of physiological data also allow the detection of processes that a participant may be unwilling or unable to report [47,48,49,50]. This, of course, applies to very young children in particular. A particular advantage of wearable sensing technology is that it enables remote continuous monitoring [1] of small or large samples, even in a familiar environment, as well as during sleep [31]. Therefore, considering the findings of the present study and the stressful and interfering nature of sleep laboratories, we propose that the use of portable ECG devices is a proper alternative to common polysomnography, especially when very young children are examined.

On the other hand, even the use of wearable sensing technology is a challenging endeavor in very young children. Although smaller than ever, the application of a wearable device in toddlers may still require a special technique of handling, and may take its time, as children usually need to feel confident to agree. In addition, the data collection always involves another (parent) person’s agreement, time, as well as compliance, which may be difficult to ensure. Finally, specific ethical aspects need to be considered and reflected from the planning of the study to its very end [51].

### 4.2. Limitations

First, 14 ECG data sets were lost, because the monitoring was stopped for some reason. This may reflect a disadvantage of wearable devices: participants’ compliance may decrease—even if data are collected passively—when they are at home and have no contact to the researcher in charge [1]. A larger sample size would have allowed the inclusion of more variables in the regression model and would have broadened the options of multivariate approaches [52]. Moreover, family income was above average in our sample, and very few children reported excessive media consumption. Low-income families are more likely to buy and use smartphones than wealthy households [53]. Therefore, the study might primarily have attracted parents that care (or worry) about their children’s media use and restrict its accessibility. This may limit the generalizability of the findings on the effects of touchscreen media use to some extent.

While the study showed high correspondence between the physiologically determined and parent-reported estimates of sleep duration, it is impossible for parents to rate the restfulness of their offspring’s sleep. Consequently, it is not possible to provide a correlation of the heart rate indicator of restfulness of sleep (*dHR*) with some parent-reported variables as an indication of the validity of its interpretation. It is a matter of further investigation how much variance the increased heart rate during restless sleep periods shares with which particular, more conventional indicators of sleep quality, gained from polysomnography in the sleep laboratory. Yet, as explained in the introduction, the use of traditional polysomnography is fraught with serious problems particularly in young children. Therefore, we propose the approach introduced in this paper as a useful alternative to polysomnography. As it can be applied in a person’s natural sleep environment (as opposed to the highly unfamiliar and sleep-interfering environment in a sleep laboratory), it may also be a good alternative to polysomnography in adults in certain instances.

## 5. Conclusions

The present results show that the expert analysis of heart rate data is a potent tool that could be used to estimate sleep quality in very young children. Additionally, in line with the recommendation that “different types of measures” [30] (p. 16) should be combined to reliably determine the quality of sleep, the additional use of a 3D acceleration sensor was able to reliably identify rest/sleep periods. Modern instruments incorporate both technologies in one small wearable device. From a technical standpoint, polysomnography remains the “gold standard” in sleep research. However, ECG recordings with additional 3D acceleration sensors, which allow estimating sleep quality in an easy and undisturbing way, may be a satisfactory and efficient alternative, particularly regarding to populations and circumstances.

## Figures and Tables

**Table 1:** Descriptive statistics of parent-reported quality of sleep.

	*n*	*M*	*SD*	Min	Max
*Nighttime sleep* (hours)	53	10.21	1.06	7.50	12.50
*Sleep onset latency* (min)	54	24.31	14.44	5.00	60.00
*Sleep time* (hours; according to activity log)	55	10.20	1.05	7.97	12.97

**Table 2:** Percentages of toddlers’ average weekly media use (*n* = 55).

Amount of Time	Never	<1 h	>1 h	>2 h	>3 h	Missing
touchscreen media	34.5	34.5	21.8	1.8		7.3
smartphone	45.5	43.6	9.1			1.8
tablet	63.6	25.5	3.6			7.3
PC/computer/laptop	87.3	7.3	1.8			3.6
TV	23.6	50.9	12.7	3.6	3.6	5.5

**Table 3:** Descriptive statistics of variables derived from the ECG.

Variables Derived from ECG Measurements	*n*	*M*	*SD*	Min	Max
*dHR sleep* (bpm)	55	7.479	3.21	−0.32	15.12
*Sleep duration* (hours)	55	10.51	0.93	8.47	12.97

**Table 4:** Results of multiple regression analyses for the analysis of the impact of touchscreen media use on the duration and restfulness of sleep.

	*Sleep Duration*				*dHR Sleep*			
	*B*	*SE*	β	*p*	*B*	*SE*	β	*p*
*Age*	−0.013	0.028	−0.068	0.655	−0.246	0.088	−0.378	0.007
*Gender*	−0.379	0.288	−0.199	0.195	−0.397	0.901	−0.059	0.661
*Touchscreen media use*	0.099	0.162	0.087	0.546	1.286	0.506	0.325	0.014

**Table 5:** Bivariate correlations between study variables.

Variables		1.	2.	3.	4.	5.	6.	7.
1.	*Age*	-						
2.	*Gender*	−0.319 *						
3.	*Touchscreen media use* ^1^	−0.026	0.096					
4.	*Nighttime sleep* ^1^	−0.075	0.058	−0.109				
5.	*Sleep onset latency* ^1^	0.243	0.084	0.165	−0.441 **			
6.	*Sleep time* ^1^	0.071	−0.257	0.036	0.162	−0.038		
7.	*Sleep duration* ^2^	0.014	−0.196	0.070	0.152	−0.048	0.867 **	
8.	*dHR sleep* ^2^	−0.357 **	0.102	0.329 *	−0.024	−0.070	−0.044	0.069

** *p* < 0.01; * *p* ≤ 0.05; ^1^ parent-reported; ^2^ based on physiological data.

## Data Availability

The data presented in this article are not available.

## Appendix A

The ECG and the body position and movement of the participants were continuously recorded using the eMotion Faros 180°. For the analysis, all raw data were converted from EDF to a MATLAB^®^ data format.

### Data Preprocessing

R-peak detection of the ECG recording was carried out with software tools of the research group based on established algorithms (e.g., [54,55]) followed by semi-automatic artifact-handling, which uses the pattern of the QRS complex and the time of occurrence within the ECG to identify “ectopic beats”. Further criteria are physiological limits and the maximal percentage of change in relation to the standard deviation of the signal on an individual basis [38].

The respiratory rate was determined offline using a modified ECG derived respiration (EDR) algorithm [38]. The algorithm also provides the rate of valid determination of the respiration pattern in the ECG signal of each recording period of each participant.

Body position and body movement were assessed by the 3D-ACC sensor, and the variance of the three channels was computed (see Figure A1d). The absolute values of the 3D-ACC sensor, which represent the body position, were used as plausibility criteria.

Variables of heart rate variability, respiratory frequency, and body position and body movement were calculated for segments of two minutes with a step size of 30 s. Selected variables, which are mandatory for the detection of restful and restless sleep phases, are shown in Figure A1b–d.

Figure A1a shows the spectrogram of the HRV based on the R–R interval using the Burg algorithm [56], to demonstrate the dominant effect of the respiration on the heart rate, which is known as respiratory sinus arrhythmia. For example, during the “afternoon nap” a dominant frequency can be seen at approximately 0.35 Hz, which represents a respiratory frequency of 21 min^−1^. However, as stated in Lackner et al. 2020 [38], time domain HRV variables should be preferred over frequency domain variables in toddlers. Therefore, the ratio of the time domain variables SDNN and RMSSD, which is SDNN/RMSSD, were used as an additional criterion.

A combinatory logic was used to define restful sleep whereby lying position (vertical axis of the 3D-ACC sensor 0 ± 0.1g relative to value at standing position), little body movement (Var_ACC_ ≤ 0.002 g), and a high percentage of valid respiratory pattern of EDR are major determinants. In addition, heart rate (change) and the ratio SDNN/RMSSD in combination with the EDR mark further possible rest phases on an individual basis. Finally, potential restful phases need a series of ten consecutive minutes, in that way determined “resting periods”, to become classified as a restful sleeping phase for further analysis. In the shown example, the percentage of restful sleep was 40.4% and the difference HR_restless_−HR_restful_ was 8.43 bpm.
